# Supplementary material for: Downregulation of Aging-Associated Gene SUCLG1 Marks the Aggressiveness of Liver Disease
Source: Cancers (Basel). 2025 Jan 21;17(3):339. doi: 10.3390/cancers17030339 (PMC11815819; doi:10.3390/cancers17030339)
Supplement: Supplementary file 1 [file cancers-17-00339-s001.zip › cancers-3368263-supplementary.pdf]

Supplementary file

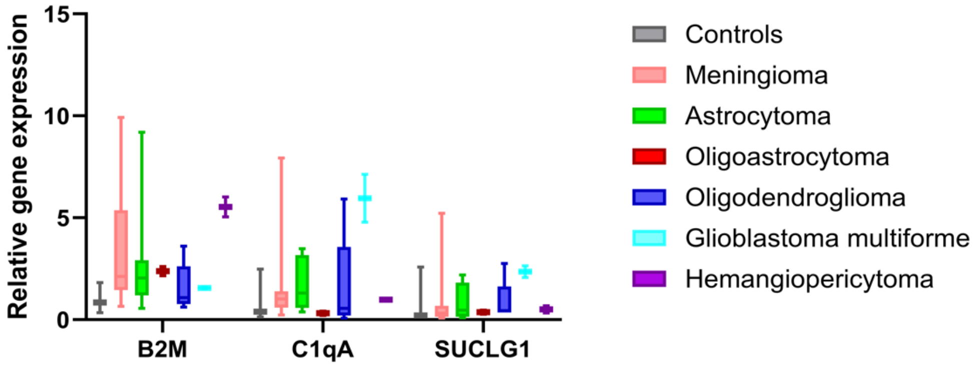

Supplementary Figure S1. Human brain cancers are not associated with changes in expression mRNA levels of B2M, C1qA, or SUCLG1. Expression mRNA levels of B2M, C1qA, and SUCLG1 in tissues with normal appearance (n=3) or tissue specimens with brain cancer alterations, associated with Meningioma (n=25), Astrocytoma (n=8), Oligoastrocytoma (n=2), Oligodendroglioma (n=5), Glioblastoma multiforme (n=2), or Hemangiopericytoma (n=2). All tissue samples in the brain cancer cohorts are biopsied from the tumor tissue area.

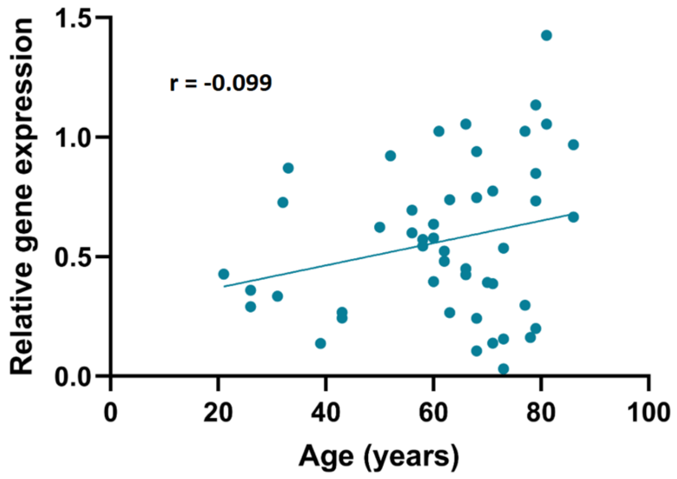

Supplementary Figure S2. SUCLG1 mRNA expression levels in liver tissue does not correlate with age. Expression mRNA levels of SUCLG1 in tissues with normal appearance (n=9) or tissue specimen with diagnosis: Fatty liver (n=5), Hepatitis (n=3),

Cirrhosis (n=5), Hepatocellular carcinoma (n=24) or Cholangiocarcinoma (n=3) were correlated with age, correlation coefficient (r) is presented on the graph.

Supplemental Table S1. Characteristics of the brain cohort patients.

Patient n	Gender	Age	Sample diagnosis	Patient diagnosis	Tumor grade
P1	Female	55	Normal	Astrocytoma	WHO Grade III
P2	Male	60	Normal	Normal	Not applicable
P3	Female	56	Meningioma	Meningioma	WHO Grade I
P4	Female	51	Meningioma	Meningioma	WHO Grade I
P5	Male	71	Meningioma	Meningioma	WHO Grade I
P6	Female	56	Meningioma	Meningioma, benign	WHO Grade I
P7	Female	41	Meningioma	Meningioma	WHO Grade I
P8	Female	42	Meningioma	Meningioma	WHO Grade I
P9	Female	73	Meningioma	Meningioma	WHO Grade I
P10	Female	64	Meningioma	Meningioma	WHO Grade I
P11	Male	39	Meningioma	Meningioma	WHO Grade I
P12	Male	43	Meningioma	Meningioma	WHO Grade I
P13	Female	83	Meningioma	Meningioma	WHO Grade I
P14	Male	68	Meningioma	Meningioma	WHO Grade I
P15	Female	45	Meningioma	Meningioma	WHO Grade I
P16	Female	61	Meningioma	Meningioma	WHO Grade I
P17	Male	44	Meningioma	Meningioma	WHO Grade I
P18	Male	61	Meningioma	Meningioma	WHO Grade I
P19	Female	54	Meningioma	Meningioma	WHO Grade I
P20	Female	63	Meningioma	Meningioma	WHO Grade II
P21	Male	67	Meningioma	Meningioma	WHO Grade II
P22	Female	56	Meningioma	Meningioma	WHO Grade II
P23	Male	73	Meningioma	Meningioma	WHO Grade II
P24	Female	48	Meningioma	Meningioma	WHO Grade II
P25	Female	71	Meningioma	Meningioma	Not Reported
P26	Female	40	Meningioma	Meningioma	Not Reported
P27	Female	46	Meningioma	Meningioma	Not Reported
P28	Female	27	Astrocytoma	Astrocytoma	WHO Grade I
P29	Female	50	Astrocytoma	Astrocytoma	WHO Grade II
P30	Male	26	Astrocytoma	Astrocytoma	WHO Grade II
P31	Female	53	Astrocytoma	Astrocytoma	WHO Grade II
P32	Male	36	Astrocytoma	Astrocytoma	WHO Grade III

P33	Male	39	Astrocytoma	Astrocytoma	WHO Grade III
P34	Male	30	Astrocytoma	Astrocytoma	WHO Grade III
P35	Female	32	Astrocytoma	Astrocytoma	WHO Grade III
P36	Female	37	Oligoastrocytoma	Oligoastrocytoma	WHO Grade II
P37	Male	41	Oligoastrocytoma	Oligoastrocytoma	WHO Grade III
P38	Male	45	Gliosis	Oligodendroglioma	WHO Grade II
P39	Male	33	Oligodendroglioma	Oligodendroglioma	WHO Grade II
P40	Male	46	Oligodendroglioma	Oligodendroglioma	WHO Grade III
P41	Female	41	Oligodendroglioma	Oligodendroglioma	WHO Grade III
P42	Female	51	Oligodendroglioma	Oligodendroglioma	WHO Grade III
P43	Female	44	Oligodendroglioma	Oligodendroglioma	WHO Grade III
P44	Male	66	GBM	GBM	WHO Grade IV
P45	Male	47	GBM	GBM	WHO Grade IV
P46	Male	63	Hemangiopericytoma	Hemangiopericytoma	Not Reported
P47	Female	66	Hemangiopericytoma	Hemangiopericytoma	Not Reported
P48	Female	49	Ependymoma	Ependymoma	WHO Grade III
